# Feasibility of combined optical coherence tomography and autofluorescence imaging for visualization of needle biopsy placement

**DOI:** 10.1117/1.JBO.25.10.106003

**Published:** 2020-10-20

**Authors:** Geoffrey Hohert, Renelle Meyers, Sylvia Lam, Andrei Vertikov, Anthony Lee, Stephen Lam, Pierre Lane

**Affiliations:** a BC Cancer Research Centre, Integrative Oncology, Vancouver, British Columbia, Canada; b University of British Columbia, Department of Medicine, Vancouver, British Columbia, Canada; c LX Medical Corporation, Westwood, Massachusetts, United States; d Simon Fraser University, School of Engineering Science, Burnaby, British Columbia, Canada

**Keywords:** optical coherence tomography, autofluorescence, biopsy, lung imaging, animal study

## Abstract

**Significance:** Diagnosis of suspicious lung nodules requires precise collection of relevant biopsies for histopathological analysis. Using optical coherence tomography and autofluorescence imaging (OCT-AFI) to improve diagnostic yield in parts of the lung inaccessible to larger imaging methods may allow for reducing complications related to the alternative of computed tomography-guided biopsy.

**Aim:** Feasibility of OCT-AFI combined with a commercially available lung biopsy needle was demonstrated for visualization of needle puncture sites in airways with diameters as small as 1.9 mm.

**Approach:** A miniaturized OCT-AFI imaging stylet was developed to be inserted through an 18G biopsy needle. We present design considerations and procedure development for image-guided biopsy. *Ex vivo* and *in vivo* porcine studies were performed to demonstrate the feasibility of the procedure and the device.

**Results:** OCT-AFI scans were obtained *ex vivo* and *in vivo*. Discrimination of pullback site is clear.

**Conclusions:** Use of the device is shown to be feasible *in vivo*. Images obtained show the stylet is effective at providing structural information at the puncture site that can be used to assess the diagnostic potential of the sample prior to collection.

## 1 Introduction

Recently, two large randomized clinical trials in the US and Europe provided solid evidence that lung cancer screening using low dose computed tomography (LDCT) of the chest can reduce lung cancer mortality by 20% to 24% in heavy ever-smokers.^1^^,^^2^ As worldwide implementation of lung cancer screening with LDCT expands, an increasing number of patients are found to have lung nodules <2  cm in diameter that requires repeat imaging studies or a diagnostic procedure.^3^ CT-guided biopsy is currently considered to be more accurate to biopsy these lesions than endoscopic biopsy but has more associated complications, such as pneumothorax and bleeding.^4^^,^^5^ A method for improving endoscopic biopsy yield could therefore reduce the overall rate of complications in diagnosing these lesions.

In transbronchial biopsies, a video bronchoscope provides visual guidance in navigating to the site of the lesion and may be supplemented by a number of other imaging modalities, such as radial endobronchial ultrasound or electromagnetic navigation, with or without fluoroscopic guidance.^4^^,^^6^^–^^9^ These technologies aide either in navigation to or confirmation of the nodule characteristics and have been shown to improve diagnostic yield. However, many imaging modalities are limited by the large size of the instrumentation relative to the airway lumen and are therefore unable to access lesions in the lung periphery and have a significantly lower diagnostic yield than endobronchially accessible lesions.^7^^,^^8^ Such peripheral lesions are common, with one study finding peripheral cancers to account for 62.7% of cases in heavy smokers.^10^ In these cases where fluoroscopic imaging is unavailable to confirm whether the target site is reached, the diagnostic yield is relatively low, potentially as a result of false negatives where the nodule tissue is actually not sampled.^11^ Therefore, a device capable of providing relevant imaging information in real time for difficult-to-access nodules could improve diagnostic accuracy for peripheral lung nodules.

Optical coherence tomography (OCT) is a volumetric imaging modality that can provide subsurface cross-sectional images of tissue structure at almost cellular resolution in real time.^12^^,^^13^ OCT has been investigated as a method of biopsy guidance in the respiratory tract by other researchers, either by classification of tissue transthoracically,^14^^,^^15^ from within the airway lumen (endobronchial),^16^^–^^19^ or by puncturing the airway wall using a needle-based OCT catheter to image nodules embedded within or located behind the airway wall (transbronchial).^20^^,^^21^

One of the main advantages of OCT for lung imaging is the relatively small size of the imaging components, making it ideal for miniaturization for high generation airway imaging and compatibility with the small working channels of bronchoscopes. OCT alone may not have the sensitivity to detect small precancerous lesions in the lung. The addition of a second imaging modality with complementary information is likely to increase the sensitivity and may enable the detection of these lesions.

We have developed a miniaturized “imaging stylet” for use with a commercially available biopsy needle, which replaces the semi-rigid nitinol stylet in the lumen of the needle. Our imaging stylet combines OCT with co-registered autofluorescence imaging (AFI). AFI provides a complementary high-resolution functional image of the structural proteins associated with precancer (collagen and elastin) and other biomarkers associated with progression to cancer including blood vessel density. The addition of AFI to white-light endoscopy in the large airways has demonstrated a six-fold increase in the sensitivity to detect precancer compared to white-light alone.^22^^–^^24^ With the OCT-AFI imaging stylet loaded into a biopsy needle, the needle can be maneuvered via video bronchoscope to the lesion site, with the aid of CT-generated computer navigation assistance, if necessary. Next, the needle may be used to puncture the suspected lesion, and then retracted to leave the imaging stylet in place to scan the tissue prior to needle aspiration. The functional biomarkers from AFI in combination with the structural OCT have the potential to improve an operators ability to confirm the placement of the needle in abnormal versus normal tissue before taking a biopsy. The scale and maneuverability of the device allow for sampling of peripheral lesions that would otherwise be difficult to access, potentially reducing the need for CT-guided biopsy.

The work described in this contribution was conducted to show the feasibility of OCT-AFI guided biopsy in small airways. Specifically, we aimed to: (1) develop an imaging catheter compatible with a commercial 18G biopsy needle; (2) develop the procedures for nodule localization, puncture, imaging, and biopsy; (3) determine if blood absorption changes the intensity of the OCT images; (4) determine if correct transbronchial images could be differentiated from incorrect endobronchial images post-puncture; and (5) determine if pre-deposited artificial nodules could be discriminated from surrounding normal tissue. We developed the imaging stylet and validated an imaging procedure *ex vivo* using resected porcine lung tissue. Image-guided biopsy was demonstrated during an *in vivo* study of three animals.

## 2 Materials and Methods

### 2.1 OCT-AFI System

We used a swept-source OCT-AFI system developed previously by our group.^25^ Briefly, a 50-kHz swept-source laser (SSOCT-1310, Axsun Technologies Inc., Billerica, Massachusetts) with 20-mW output power feeds a single-mode fiber 90/10 sample/reference split Mach–Zehnder OCT interferometer. Light for fluorescence excitation from a 445-nm semiconductor laser is coupled into the core of the polyimide coated double-clad fiber (DCF, FUD-3489, Nufern, East Granby, Connecticut) with the OCT light using a wavelength division multiplexer. The sample arm consists of a fiber-optic rotary joint that connects the stationary optics to the rotating core of the imaging stylet. A custom-built rotary-pullback drive allows rotation speed up to 33 Hz and pullbacks up to a maximum length of 160 mm performed at 1  mm/s. The OCT interferogram is acquired using a fast digitizer (ATS9350, Alazar Technologies Inc., Pointe-Claire, Quebec) in “k-clock” acquisition mode with custom data acquisition software. Autofluorescence light is collected in the inner cladding of the DCF, detected by a photomultiplier tube (H10723-20, Hamamatsu, Japan), and digitized by the second channel of the fast digitizer.

The OCT modality has a measured resolution of 24 um in the longitudinal direction and 14 um in the circumferential direction, and an imaging depth of ∼1 to 2 mm in tissue. The AFI modality has a measured spot size at the surface of the stylette of 18 um in the longitudinal direction and 12 um in the circumferential direction. The AFI beam does not penetrate more than 1 mm into tissue and its spot size increases quickly with distance from the imaging stylet.

### 2.2 OCT-AFI Imaging Stylet

The needle-compatible imaging stylet is based on an existing OCT-AFI catheter design developed by our group. The design was modified to be compatible with the commercial biopsy needle (FleXNeedle, 18G, Broncus Medical Inc, San Jose, California) selected for this work. The stylet included with the biopsy needle had an outer diameter (OD) of 0.69 mm. The OD of our existing catheter had to be reduced from 0.90 mm to match and pass easily through the needle lumen. Additionally, the insertable length of the catheter design was increased from 130 to 175 cm to span the entire length of the needle. The entire device was deployed through the instrument channel of a standard bronchoscope (Olympus BF Type P180A—EVIS EXERA II bronchovideoscope).

By changing the diameter and length of the catheter, mechanical performance can be impacted, as characterized by non-uniform rotational distortion (NURD) at the distal tip of the rotating optical assembly. Imaging stylets were tested in 3D printed channels with eight equally spaced markers placed along their length to highlight any deviations in a scan pattern from expected patterns.^26^ This was done both with the imaging stylet unconstrained and contorted to match a typical path it would take through a bronchoscope and into human lung.

For the reduced-diameter window tube, Pebax^®^ 70D, Pebax^®^ 72D, and PET were considered and tested as potential tube materials due to their optical clarity and flexibility.

**Fig. 1:**
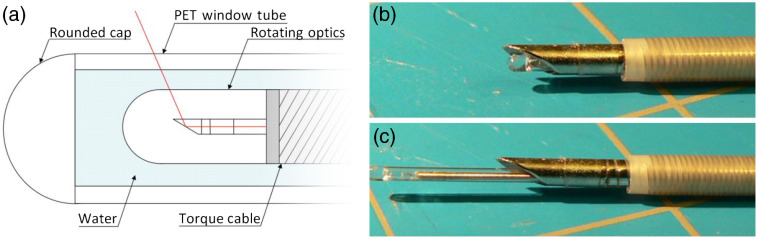
Distal end of the imaging stylet. (a) Schematic showing the OCT and excitation light path in red. (b) Imaging stylet loaded in the biopsy needle and retracted slightly for puncture and (c) stylet extended for imaging.

The distal tip of the imaging stylet is shown in Fig. 1.

To reduce the OD of the stationary window tube to fit inside the biopsy needle, the diameter of the rotating optical assembly needed to be reduced to maintain clearance between the two. The torque cable [Fig. 1(a)] determines the size of the rotating assembly and runs the length of the probe, protecting the optical fiber while delivering the proximally-driven torque to the distal tip. A high torsional stiffness is desirable to avoid NURD^27^ but often comes at the cost of decreased flexibility that limits the assembly’s ability to perform well in low-radius bends when conforming to lung anatomy. We ordered and tested custom double-wound torque cables (Asahi Intecc Co. Ltd., Aichi, Japan) with an OD of 0.33 mm and an inner diameter of 0.17 mm. Measurements made to estimate bending and torsional stiffness of the cable were both significantly lower than existing cables, but measurements of NURD performance showed torque cable-induced distortion comparable to our existing probes in straight orientations, and slightly superior in tortuous conditions emulating the path the bronchoscope would take in clinical conditions.

The stationary window tube [Fig. 1(a)] surrounds the rotating optical assembly and the water that acts as lubricant and optical coupling medium to reduce reflections due to index of refraction changes. The window tube outer surface makes contact with the entire inside length of the biopsy needle and tissue, when extended. Optical clarity, low friction, and resistance to buckling are all requirements of acceptable tubing. Of the materials tested, Pebax^®^ offered better resistance to buckling, whereas PET is stiffer and therefore, more easily passed through the narrow lumen of the biopsy needle (i.e. higher “pushability”). A probe was constructed using Pebax^®^ 70D, which was found to stick inside the biopsy needle occasionally despite having low friction over small distances. Another probe was made using PET to compare and was found to not stick even when the needle was contorted into tortuous orientations to simulate clinical conditions. The PET tubing was used for all future window tubes.

**Fig. 2:**
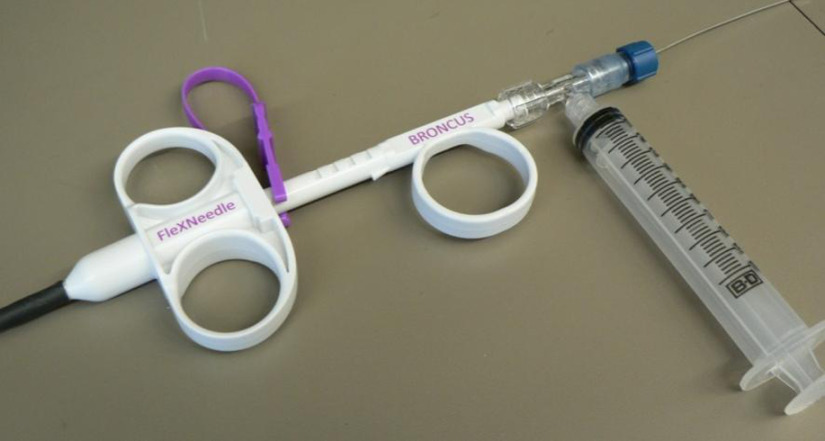
The proximal handle of the Bronchus FleXNeedle, with attached T-junction for vacuum suction, and releasable luer seal around the imaging stylet.

For flushing water, a T-junction luer lock connector was added to the proximal end of the biopsy needle handle (Fig. 2), such that a seal could be formed around the imaging stylet and water irrigated between the stylet and needle using an attached syringe. This also allowed for the application of vacuum pressure while the imaging stylet was still loaded (but partially retracted) in the needle to collect aspiration biopsy samples.

### 2.3 Image-Guided Needle Puncture Procedure

**Fig. 3:**
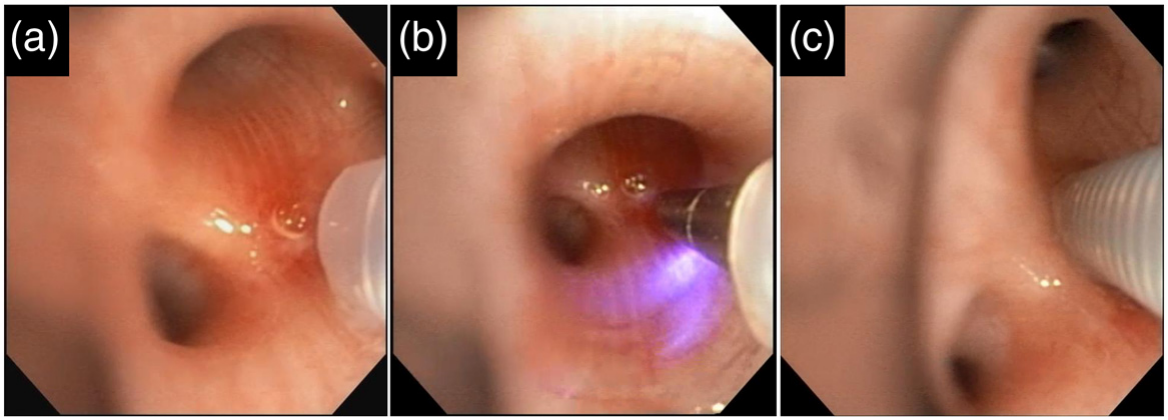
Videobronchscope images of the needle being (a) maneuvered into view; (b) advanced out of the tubing by 1 cm; and (c) used to puncture the airway wall.

**Fig. 4:**
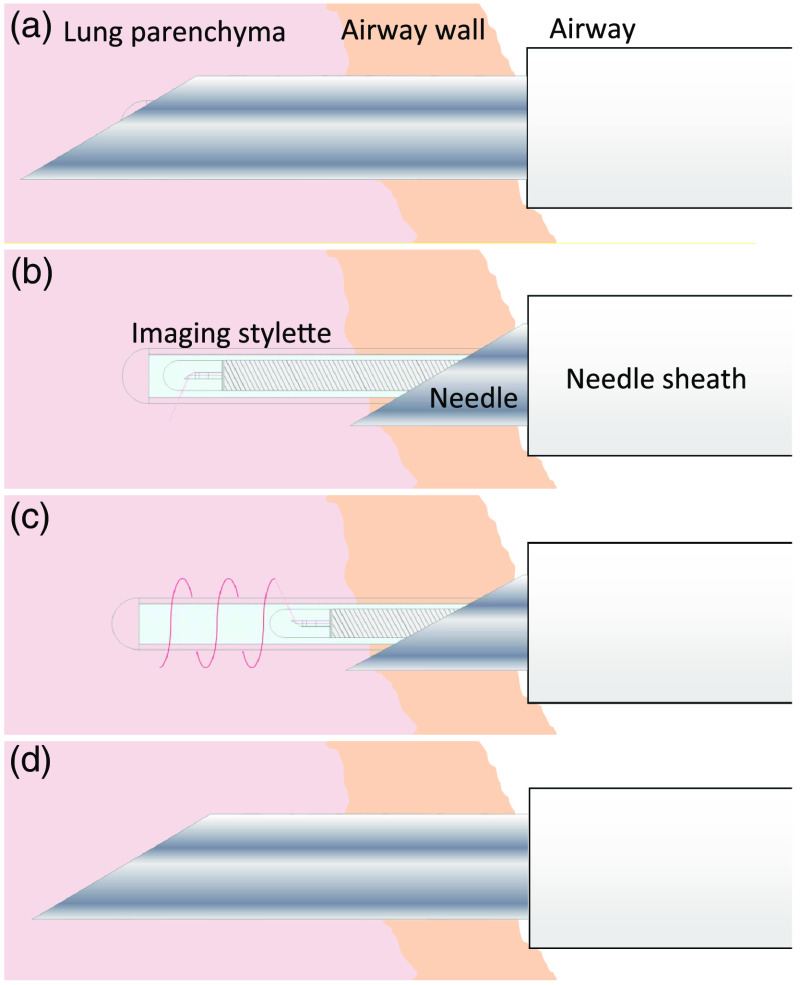
Puncture and pullback procedure. The needle and imaging stylet puncture the tissue together (a) before the needle is retracted by 10 mm while the stylet is held in place (b). The stylet is then used to capture a helical scan of the punctured tissue (c) and the needle is then re-advanced over the stylet into the tissue being scanned (d), at which point the stylet can be withdrawn from the needle for TBNA.

**Fig. 5:**
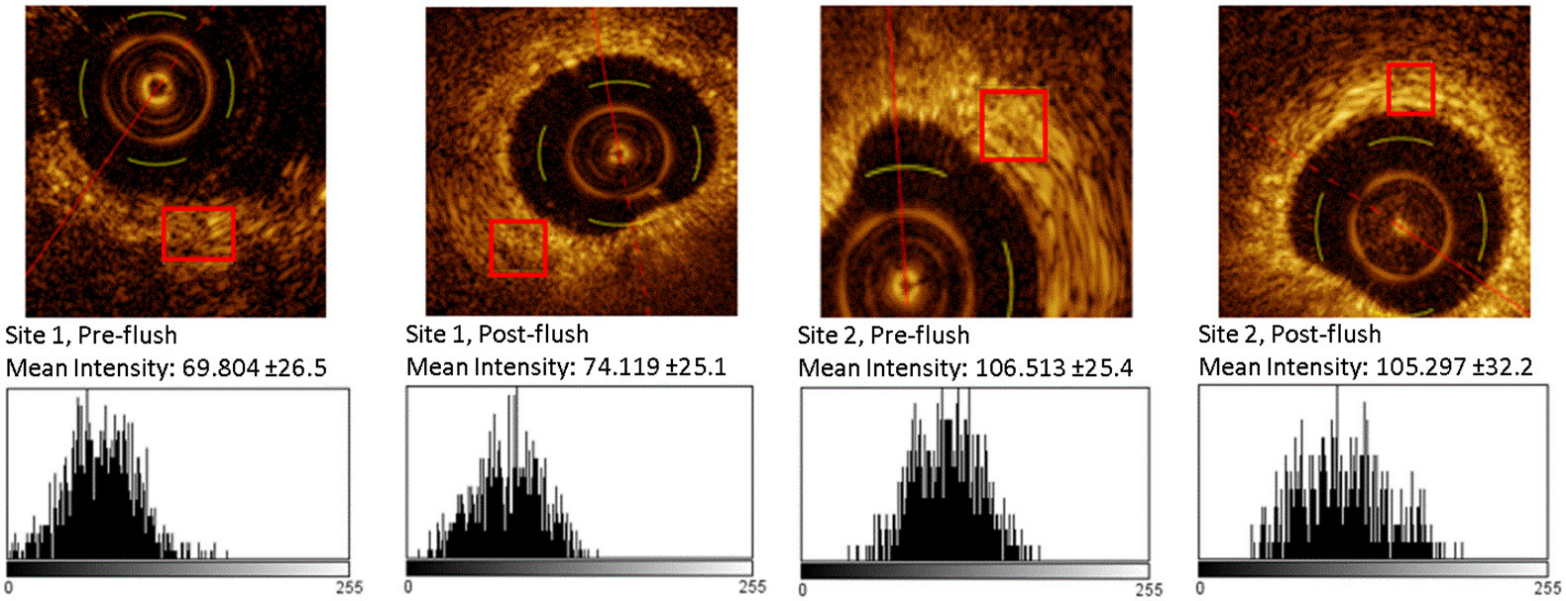
Pre and post-flush histograms of the OCT signal. Histograms were calculated from pixels in the red boxes, with the mean intensity shown at each site, and standard deviation in parentheses. There was <5 gray level difference between the mean OCT signal at site 1 and <2 gray level difference at site 2, indicating minimal improvement in penetration and contrast.

**Fig. 6:**
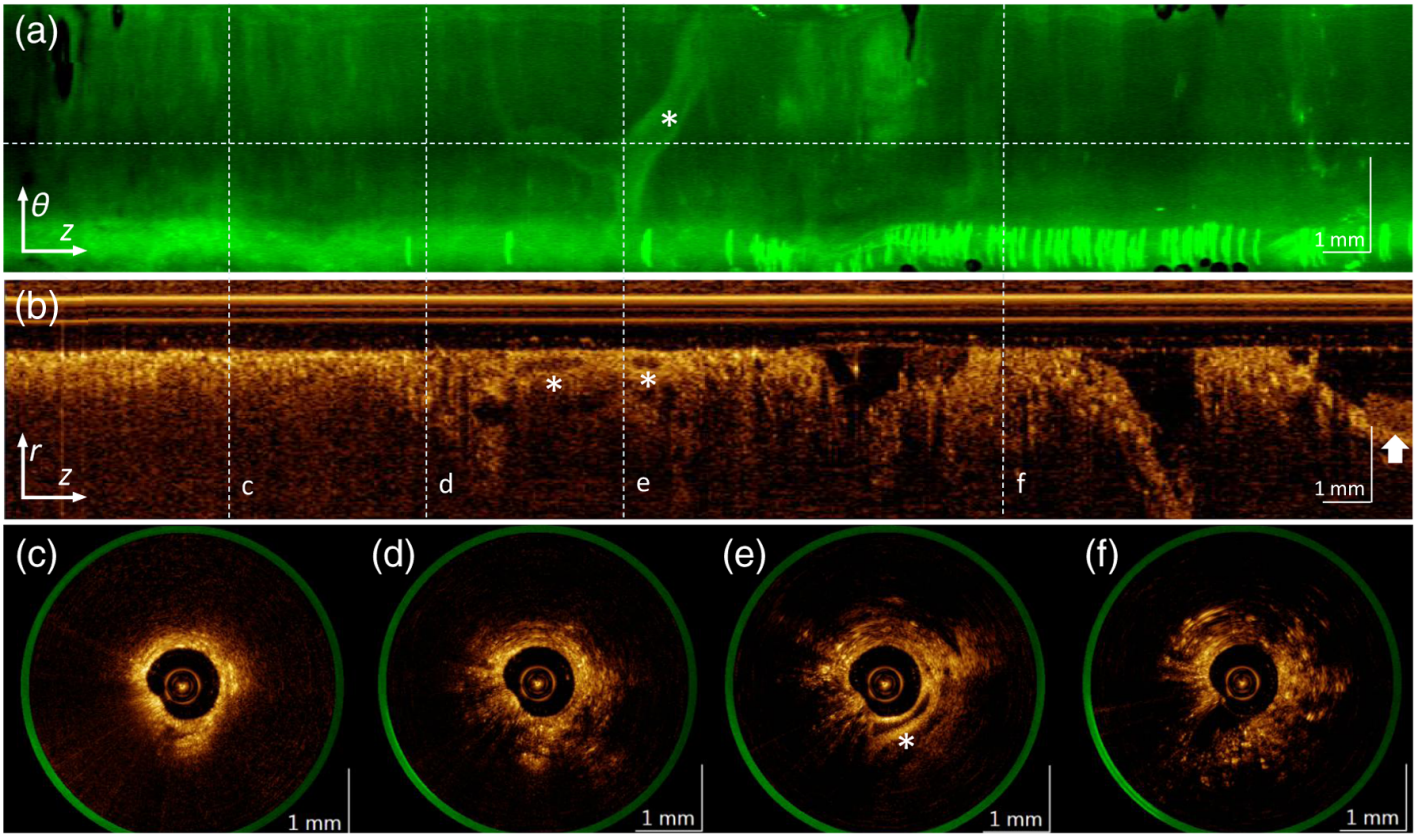
Example transbronchial pullback from an airway in the cranial right lobe showing a blood vessel, with the distal end of the scan on the left: (a) en-face AF projection; (b) OCT azimuthal pullback showing depth into tissue at the angle marked by the horizontal dashed line in (a); and four cross-sections (c)–(f) taken at the positions marked with vertical dashed lines. A vessel is visible in the AF projection, and in cross section in (b) and (e), marked with *. The needle outer tube is also visible at the proximal end of the scan, indicated with an arrow. The horizontal band with intense vertical streaks at the bottom of the AFI image is an artifact.

**Fig. 7:**
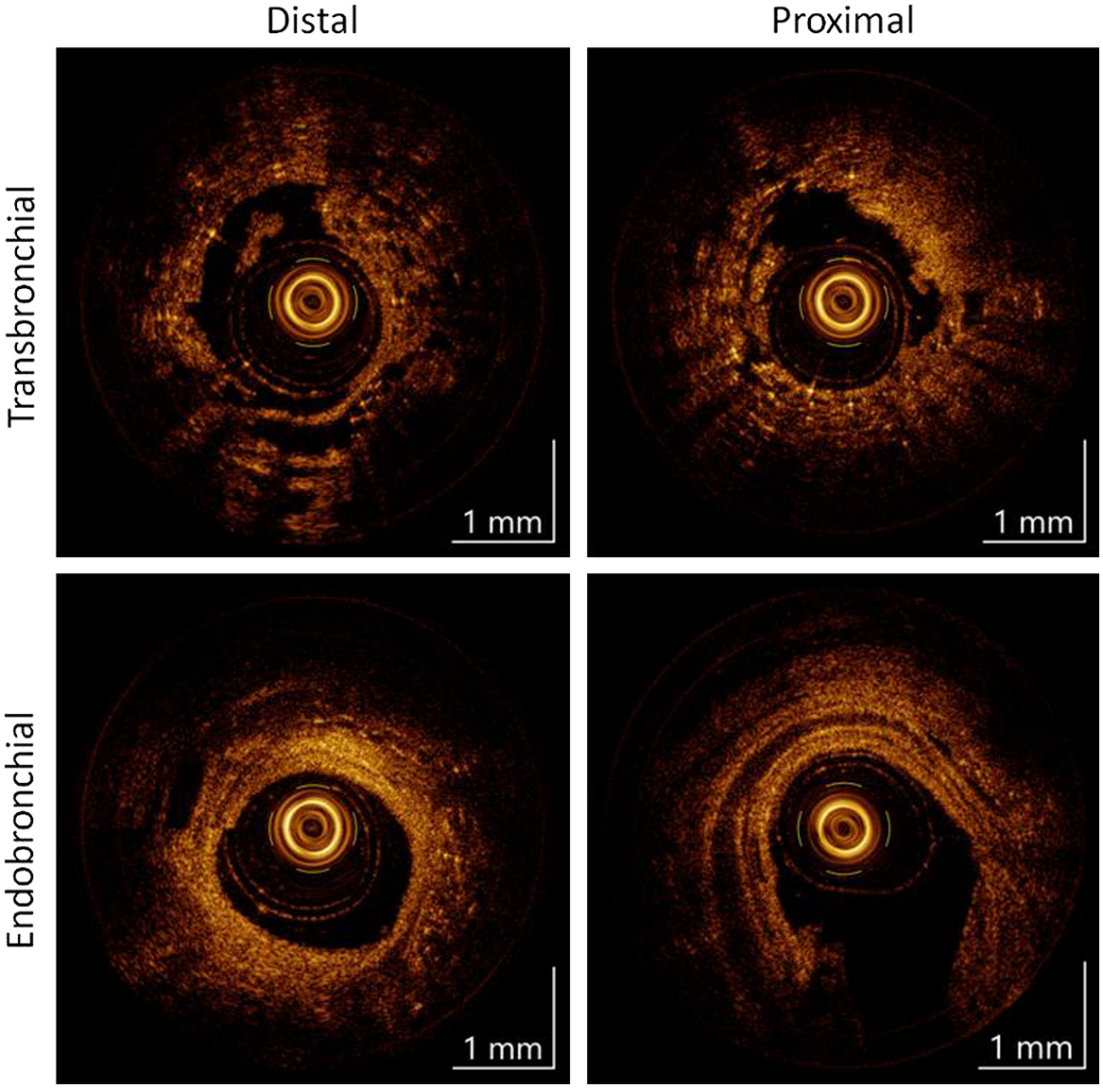
Cross-section comparison of OCT features observed in transbronchial and endobronchial imaging: (a) transbronchial distal scanning shows alveoli in most directions, and only a thin membrane separating the probe from alveoli; (b) transbronchial proximal scanning still shows some alveoli, but also the more solid tissue of airway wall in the upper right; (c) endobronchial distal scanning shows some alveoli, but separated from the probe by airway wall or supportive tissue; and (d) endobronchial proximal scan showing an airway much larger than the probe, with CRs concentric with the airway. CR: cartilage band.

The protocol for image-guided biopsy was developed through *ex vivo* experimentation with porcine lung tissue. This led to the following puncture and pullback protocol (with reference to Figs. 3 and 4), which was followed during the subsequent *in vivo* imaging studies: (1) navigate the bronchoscope (with needle preloaded) to the desired airway; (2) verify bronchoscope position with fluoroscopy; (3) extend 10 mm of needle from the bronchoscope and angle the needle tip toward the desired site; (4) puncture by advancing the needle forward such that the needle penetrates the airway wall by 10 to 20 mm (Fig. 3); (5) irrigate the puncture site with saline [Fig. 4(a)]; (6) retract only the needle, leaving the imaging stylet in place [Fig. 4(b)]; (7) collect a pullback with imaging stylet [Fig. 4(c)]; and (8) re-advance the needle around imaging stylet [Fig. 4(d)].

Where biopsy aspiration is indicated by image confirmation of a diagnostic site, the imaging stylet may then be fully retracted such that transbronchial needle aspiration (TBNA) can be performed at the same site.

Sites in the cranial right lobe were punctured to a depth of 10 mm. Sites were flushed with sterile water prior to imaging to displace blood, but two sites were imaged immediately after puncturing and prior to flushing to compare image quality. Figure 5 shows cross-sectional B-scans taken from pre- and post-flushed puncture sites with histograms of OCT intensity values for subsets of the image at roughly the same depth into tissue. The change in mean OCT intensity at site 1 (<5 gray levels, 1.6%) is significant (P=0.007), whereas the change at Site 2 (<2 gray levels, 0.6%) in not significant (P=0.5082).

### 2.4 *Ex Vivo* Imaging of Needle Puncture Site

The operation of the imaging stylet with the commercial biopsy needle was validated using six non-inflated excised porcine lungs. Organs were donated by researchers at the UBC Centre for Comparative Medicine after conducting unrelated studies covered by existing Animal Care protocols and ethics approvals.

Three lungs were also acquired and inflated using both positive internal and negative external pressure to inflate the alveoli as much as possible and mimic the OCT-AFI appearance of peripheral lung tissue *in vivo*.

In both cases, we navigated to the lung site of interest using the bronchoscopes white-light video and pierced with the needle up to a depth of 20 mm. Next, the needle was retracted fully into the needle sheath while the stylet and the needle sheath were kept stationary. The stylet, now embedded in tissue, was used to scan the length of the puncture. These pullbacks were acquired at 33 Hz, 1  mm/s over 90-mm total length. AFI gain for each case was adjusted to achieve the highest contrast between the brightest and darkest features in a representative pullback.

### 2.5 *In Vivo* Imaging of Needle Puncture Site

Image-guided biopsy was performed on three live swine using the commercial biopsy needle and the OCT-AFI imaging stylet during bronchoscopy based on standard instructions for use of transbronchial biopsy needles.^28^^,^^29^ Procedures were performed in the Animal Care Facility at the Jack Bell Research Center and the study was approved by the UBC Animal Care Committee (A16-0029). The swine were sedated with acepromazine and ketamine followed by intravenous propofol. Animals were intubated and ventilated with blood pressure, $O_{2}$ saturation, and heart rate monitored throughout the procedure.

Owing to anatomic differences (swine have longer trachea and airways that branch more slowly compared to humans^30^), image-guided biopsy was performed in the cranial lobe as the other lobes were out of reach using a human bronchoscope. The cranial lobe branches from the trachea just prior to the main carina, and as such, involves a sharp turn similar to upper-lobe anatomy in the human lung. At each biopsy site, after puncture with the needle, sterile water was flushed around the stylet through the aspiration channel. Flushing was done to displace any blood present at the site, which may otherwise reduce penetration of OCT light and absorb the autofluorescence excitation and/or emission light. At two locations, pullbacks were acquired before and after flushing to compare image quality and inform whether regular flushing is necessary. The mean OCT intensities from pullbacks collected before and after a saline flush were compared using a two-tailed student’s t-test.

### 2.6 Discrimination of Trans- and Endobronchial Pullbacks

Endobronchial pullbacks were collected by navigating the biopsy needle into the lumen of the target airway, advancing the imaging stylet from the needle, maneuvering the imaging stylet such that it was in contact with the airway wall along the desired pullback length, followed by acquisition of the pullback. Transbronchial pullbacks were collected by puncturing the airway wall (with stylet retracted), retracting the needle leaving the stylet in the puncture site, followed by acquisition of the pullback. OCT and AFI features were identified in the pullbacks that could discriminate between the endobronchial and transbronchial pullbacks. Endobronchial pullbacks were collected in RB1Cr and RB1Cd (cranial lobe) and compared with transbronchial pullbacks collected from puncture sites in the same airway.

### 2.7 Nodule Confirmation

Following a similar protocol for the transthoracic deposition of artificial nodules in human lungs *in vivo* performed by Tsuchida et al.,^31^ powdered agarose and acridine orange were dissolved at 1.5% and 0.002% w/v, respectively, in water at 60°C. When cooled to body temperature, this mixture yields a solid mimicking the stiffness of lung parenchyma. The concentration of acridine orange was chosen to slightly exceed the highest level of fluorescence expected in normal lung tissue. Artificial fluorescent nodules could then be created by injecting the agarose/acridine orange/water mixtures into the parenchyma of one swine using an 18G needle designated for nodule placement.

Nodule deposition and detection were performed *in vivo* following process development on one of the *ex vivo* porcine lungs. We created two nodules in the cranial lobe and two nodules in a lower lobe.

At each nodule site, we first collected a transbronchial OCT pullback as a negative control. The solution and delivery needle were kept at 60°C until immediately prior to injection to keep the mixture fluid. The warmed needle, loaded with ∼0.5  ml of the agarose solution, was advanced quickly down the bronchoscope and used to puncture the airway at a depth of 5 mm. Positive pressure at the proximal port was applied and held for 30 s to allow the semi-viscous agarose to flow into the tissue. The needle was then unloaded from the bronchoscope and prepared for the next site while the nodule was left for at least 2 min to set before attempting to re-puncture and collect a second pullback for nodule detection. The bronchoscope was kept in position throughout to keep the puncture site in view of the white-light camera so that the needle could more accurately be maneuvered to the same airway wall section that was just punctured.

## 3 Results

### 3.1 Transbronchial Pullback

A transbronchial pullback from an airway in the cranial lobe is shown in Fig. 6. The autofluorescence information from the pullback is shown as a θz surface plot [Fig. 6(a)] since AFI is not depth-resolved. Volumetric OCT information is plotted in longitudinal cross sections, r-z [Fig. 6(b)] and circular cross-sections, θr [Figs. 6(c)–6(f)].

Figure 6(a) shows the AFI plot where the mucosa at the distal (leftmost) end of the puncture site is non-fluorescent and appears dark, and a 0.4-mm diameter blood vessel is seen forking at Fig. 6(e). The bright horizontal broadband at the bottom of the AFI plot with brighter vertical streaks increasing in number toward the right is an artifact due to specular reflections of the excitation light from the window tube being coupled back into the imaging catheter. This artifact was observed at most sites in the cranial lobe owing to the rotating optical assembly resting non-coaxially within the window tube in the non-rigid puncture cavities.

The OCT longitudinal cross section [Fig. 6(b)] shows the depth information at the horizontal cut (dashed white line) indicated in the AFI plot. The needle sheath is visible on the right side of the pullback (indicated by the white arrow). The vertical white lines indicate the position of the four cross-sections in the bottom panels. Figure 6(c) shows the probe entirely embedded in homogenous structural tissue, with no significant adjacent features, compared to Fig. 6(d), which has a grainier appearance suggesting unfilled alveoli from nearby airways. Figure 6(e) shows the medium-sized vessel near the puncture (indicated with an asterisk), and alveoli that are filled with air (dark semi-round cavities, especially on the left side).

OCT image quality, which is somewhat reduced due to multipath interference (ghosting) in DCF-based multimodal OCT systems, is comparable to the quality observed in our larger-diameter OCT-AFI catheters upon which the smaller-diameter imaging stylet is based. Features such as alveoli, mucus, airway epithelium, and adjacent airways are all clearly discernible. The needle sheath is visible at the end of the pullback, providing a physical reference point for distance measurements.

In the AF images, alveolar patterns are visible in the smaller distal airways, and superficial blood vessels of various sizes can be traced in some cases. We see a relatively uniform distribution of tissue autofluorescence, owing to the structural proteins collagen and elastin that normally provide mechanical support for the airways.

### 3.2 Discrimination of Trans- and Endobronchial Pullbacks

Figure 7 compares cross-sectional imaging from transbronchial and endobronchial pullbacks at distal and proximal locations taken from non-inflated excised porcine lungs. At the distal position, the transbronchial cross-section shows homogenous backscatter that gradually decreases in intensity, whereas the endobronchial cross-section shows stratified intensity with bright structural tissue around the stylet more sharply transitioning to less scattering tissue, and alveoli present and separated from the stylet. At the proximal position, the transbronchial cross-section still has tissue surrounding the stylet, with minimal structure and bright features (possible un-filled alveoli) distributed both in contact with and separated from the stylet; in the endobronchial cross-section, the stratified tissue structure has expanded smoothly to reveal a lumen larger than the stylet, and a structural cartilage band (CR) is visible and concentric with the airway.

### 3.3 Nodule Confirmation

We deposited four artificial nodules in one of the swine and then attempted to identify the nodules based on their fluorescent contrast two minutes later after the nodules had solidified.

The time between readying the solution and injection varied only by about 10 to 30 s for each nodule, but it was apparent that some nodules were fluid enough to leak out of the puncture site, whereas others solidified too much in the needle to be ejected. Another complication was the heat from the agarose needle caused the bronchoscope lens to fog up (despite using anti-fogging solution), which made confirmation of nodule deposition and relocation of the nodule site even more difficult.

**Fig. 8:**
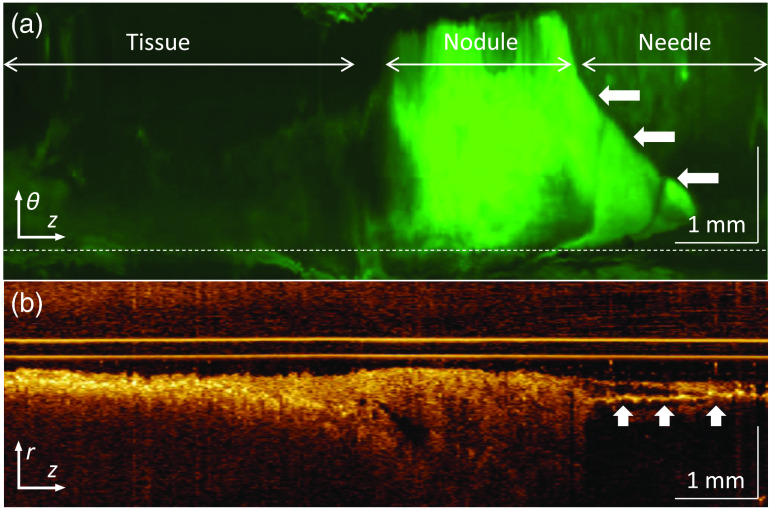
Transbronchial pullback through an artificial lesion: (a) enface AFI and (b) OCT azimuthal pullback showing depth into tissue at the angle marked by the horizontal dashed line in (a). The distal (left) half of the pullback is in normal tissue, followed by the bright positive-fluorescence artificial lesion, and finally the needle (indicated with arrows in both views).

One of the four artificial nodules provided a strong AFI signal, one provided a weak signal, and two nodules provided no signal above the background level of normal tissue autofluorescence. The puncture site that returned the clearest response is shown in Fig. 8, with the nodule located on the right, visible as a brightly fluorescent mass in the enface AFI image [Fig. 8(a)], and a semi-homogenous layer in OCT [Fig. 8(b)], with lower-fluorescing “normal” tissue in the distal direction. Differentiation of tissue and lesion in the figure is possible in OCT but much clearer in AFI. The needle becomes visible on the proximal side of the scan, obscuring tissue on the proximal side of the nodule in both the AFI and OCT images. As such, the imaging stylet was able to positively identify whether a nodule had been punctured with a single 20 second-long scan in one of the four attempts.

## 4 Discussion

OCT has been used extensively for imaging, and has been hailed as having the potential to act as an “optical biopsy,”^12^^,^^14^^,^^17^ replacing the need for excision and histopathology with real-time imaging of comparable resolution, but efforts to establish OCT as a standalone replacement for the current gold standard are still ongoing. While the technology continues to drive improvements in OCT toward this goal, co-registered imaging modalities provide a more immediately attainable upgrade in image utility. For example, Hariri et al.^32^ have reported on their polarization-sensitive OCT system enabling “accurate detection of tumor-associated fibrosis” in excised lung tissue, with the intention of improving lung biopsy yield.

AFI can provide additional functional biomarkers that may improve the sensitivity of OCT-AFI compared to OCT alone. The reduced density of structural proteins, particularly collagen and elastin in the stroma, is an established indicator of precancerous progression in the epithelium. Studies in the lung and cervix have shown that reduced protein density reduces the fluorescent intensity detected by AFI. Precancerous lesions that appear dark under AFI are likely to be more advanced that brighter ones, and a biopsy as this location may improve the diagnostic yield compared to collecting a biopsy from adjacent tissue where the AFI signal is higher. Further, AFI may also enable the assessment of vessel density, which may further improve the sensitivity to detect malignant lesions.

Similar devices to the one reported here have been developed and validated by other researchers. Tan et al.^21^ developed a 21G ball-lens OCT “smart needle” and tested it on inflated excised pig lung. Michel et al.^19^ described an *in vivo* human lung pilot study where biopsy is taken by a separate instrument at the location indicated by OCT. Our addition of AFI to this class of endoscopic biopsy guidance demonstrates that the information provided by OCT can be supplemented by adjunctive image modalities without sacrificing size or compatibility with existing flexible biopsy needles. However, the addition of AFI to OCT devices requires the use of DCFs, which introduce significant and difficult to manage noise into the OCT image. While future fiber design improvements may reduce image degradation, this tradeoff is still expected to be advantageous in clinical cases where OCT is not necessarily diagnostic on its own but useful for the interpretation of the added imaging modalities.

We also add to the limited number of *in vivo* cases performed with OCT-assisted flexible needle imaging studies, specifically confirming that blood collection is not a major concern in living tissue: In previous studies this lab has performed, imaging sites have been flushed with saline to prevent blood from absorbing OCT and AFI light and reducing contrast and depth penetration. We expected that puncturing into transbronchial tissue would result in a blood-occluded scan but found that the scans were not degraded by the presence of blood enough to interfere with differentiation of trans- and endobronchial scan locations, nor was structural information more difficult to trace for accurate interpretation of AFI.

Endobronchial “punctures” were another practical concern that was addressed after we determined that about 1 in 3 *ex vivo* punctures extended into adjacent airways. Such a puncture in *in vivo* tissue would be greatly misleading (in terms of informing what tissue had been punctured) if they could not be distinguished using the images obtained. We found that endobronchial pullbacks could be differentiated from transbronchial scans by identifying any number of the puncture-specific features described in Sec. 3.4 of this paper. OCT texture and structure were consistent with previous imaging studies comparing punctured parenchyma and intact airway.^33^ Additionally, branch points (not shown) are easily identified in the enface projection of a pullback and indicate endobronchial placement.

Although verifying transbronchial puncture would be important in human clinical cases, it is possible that endobronchial punctures are only of frequent concern in porcine lung anatomy. This is due to porcine airways branching and running roughly parallel to each other, whereas human airways tend to bifurcate at higher angles, especially in the periphery.^30^ Not inflating the excised lungs may also have contributed to the frequency of endobronchial punctures.

Regarding the final aim of simulating nodules to demonstrate image verification at a site of interest, we encountered a number of practical issues. Producing stable, contained nodules in the porcine airway required a concentration of agarose that was fluid enough to be ejected from the needle while being viscous enough to keep its shape under pressure before gelling completely. Given the difficulty in maintaining viscosity and the strong AF response from even small amounts of the agarose solution, we suspect that placement and/or re-puncturing of the nodules failed in the two low-signal cases, rather than the OCT-AFI stylet failing to detect the artificial nodule. More reliable deposition methods are required to determine the reliability of OCT-AFI to discriminate nodule characteristics, but the single successfully identified nodule shows that the procedure is feasible.

One of the most significant improvements that could be made to the device is allowing imaging of the biopsy site as the biopsy is being taken to remove any uncertainty that the sample obtained might not be from the area of interest. A number of imaging modalities already exist for other organ sites that can visualize tissue as it is being collected, and represent one of the highest levels of certainty in obtaining diagnostically relevant biopsies. Additionally, biopsy locations with even smaller diameters may require a device with a further reduced OD.

For future applications, the miniaturized OCT-AFI catheter lends itself to use as an accessory imaging tool in cases where space is limited and other visualization methods cannot be used, particularly in luminal organs such as the pancreas. Additionally, although we have used the co-registered channel for 445-nm autofluorescence, the channel may also be used for reflectance imaging, or fluorescence imaging of dyes or markers, depending on the site and tissue characteristics that are most likely to inform confirmation of the target tissue characteristics.

## 5 Conclusion

We have developed an imaging stylet that can be deployed through a commercial biopsy needle to allow image confirmation of tissue characteristics at the biopsy site. We demonstrated transbronchial lung biopsy using *ex vivo* and *in vivo* porcine lung. Integration into a commercial biopsy needle procedure was straightforward and did not add significant time or risk of complications. This study has shown that it is practical and feasible to add OCT-AFI to conventional biopsy procedures with the aim of improved diagnostic yield. The quantitative improvements in diagnostic yield of transbronchial needle biopsy due to the inclusion of OCT-AFI remain to be shown. To the best of our knowledge, we present here the first *in vivo* demonstration of a flexible transbronchial OCT-assisted biopsy device.
